# The America I Know Could Use a Good Cry: Impact of State and Civilian‐Perpetrated Racial Violence on Cognitive Health

**DOI:** 10.1002/alz70860_096609

**Published:** 2025-12-23

**Authors:** Muriel Taks Calle, Betselot Wondimu, Cailyn Clemons, Paris AJ Adkins‐Jackson

**Affiliations:** ^1^ Columbia University, New York, NY, USA; ^2^ Columbia University Mailman School of Public Health, New York, NY, USA

## Abstract

**Background:**

Incarceration in the United States (U.S.) is a structural determinant of adverse biological aging. Given the interplay of biological and cognitive health in neurodegeneration leading to Alzheimer's disease, there is a need for research exploring the relationship between incarceration and cognitive health.

**Method:**

County‐level jail and prison incarceration rates in the nearly decade‐long period preceding the passing of the 1994 Crime Bill were linked to cognitive test performance scores from the Health and Retirement Study (HRS). The analysis focused on participants racialized as Black, aged 51 or older who completed HRS core interviews on their own, and did not present dementia at baseline (*N* = 185). The ratios of incarcerated individuals racialized as Black to the total jail/prison population per county were included as explanatory variables. If this ratio was ≥.5 at any time between 1985‐1994, the county was coded as 1 to indicate structural racism. The dependent variable was assessed using the Langa‐Weir classification. Participants were coded as 1 if their test scores indicated cognitive impairment (7‐11) or dementia (0‐6) at any time between 2008‐2020. The initial sample was filtered to include those participants racialized as Black living in U.S. counties where at least one person racialized as Black was lynched between 1877‐1950 and with racially disproportionate police violence between 1985‐1994. Binomial logistic regressions estimated the association between living in a county with disproportionate Black jail and prison incarceration and cognitive impairment.

**Result:**

Participants racialized as Black living in a county with a history of lynching, disproportionate police killings, and disproportionate jail and prison incarceration were 2.8 (95%CI [‐0.003, 5.834]) times more likely to have at least one incident of cognitive impairment between 2008‐2020 compared to participants racialized as Black living in a county with a history of lynching and disproportionate police killings but not disproportionate incarceration.

**Conclusion:**

Enduring regional racial violence increases the chances of experiencing cognitive impairment among individuals racialized as Black. It is feasible that such individuals experience cumulative stressful and fear‐related experiences that are detrimental to their health across the life course.

